# Beyond cells – The virome in the human holobiont

**DOI:** 10.15698/mic2019.09.689

**Published:** 2019-07-01

**Authors:** Rodrigo García-López, Vicente Pérez-Brocal, Andrés Moya

**Affiliations:** 1 Institute of Evolutionary Systems Biology (I2Sysbio), Universitat de València and CSIC, València, Spain.; 2 CIBER in Epidemiology and Public Health (CIBEResp), Madrid, Spain.; 3 Fundación para el Fomento de la Investigación Sanitaria y Biomédica de la Comunidad Valenciana (FISABIO), València, Spain.

**Keywords:** viral metagenomics, bacteriophages, microbiota, databases, taxonomy

## Abstract

Viromics, or viral metagenomics, is a relatively new and burgeoning field of research that studies the complete collection of viruses forming part of the microbiota in any given niche. It has strong foundations rooted in over a century of discoveries in the field of virology and recent advances in molecular biology and sequencing technologies. Historically, most studies have deconstructed the concept of viruses into a simplified perception of viral agents as mere pathogens, which demerits the scope of large-scale viromic analyses. Viruses are, in fact, much more than regular parasites. They are by far the most dynamic and abundant entity and the greatest killers on the planet, as well as the most effective geo-transforming genetic engineers and resource recyclers, acting on all life strata in any habitat. Yet, most of this uncanny viral world remains vastly unexplored to date, greatly hindered by the bewildering complexity inherent to such studies and the methodological and conceptual limitations. Viromic studies are just starting to address some of these issues but they still lag behind microbial metagenomics. In recent years, however, higher-throughput analysis and resequencing have rekindled interest in a field that is just starting to show its true potential. In this review, we take a look at the scientific and technological developments that led to the advent of viral and bacterial metagenomics with a particular, but not exclusive, focus on human viromics from an ecological perspective. We also address some of the most relevant challenges that current viral studies face and ponder on the future directions of the field.

## HIDDEN IN PLAIN SIGHT – VIRUSES IN A MICROBIAL WORLD

As of this century, it is well accepted that humans are not alone in their bodies but are, in fact, hosts to a remarkably complex microscopic ecosystem comprised by a vast and thriving community of viruses, bacteria, archaea, fungi and other eukaryotes, collectively referred to as the human microbiota [1]. Science has undeniably come a long way since this miniscule, yet lively, inner world was first observed under Leeuwenhoek's skillfully crafted microscopes back in the 17^th^ century [2] but our understanding is far from complete as the scope of microbiota research continues expanding. We know that under normal conditions, each subject holds an astonishing variability of microbial agents (over a thousand different prokaryotic species have been successfully characterized from the healthy adult human gastrointestinal tract, an interconnected system comprising the most profusely populated microbial niche in humans [3]). Furthermore, these microscopic agents are not just foreign and pathogenic in nature, a long-standing misconception held until the second half of the 20^th^ century, but they are actually ubiquitous and some even potentially advantageous for their human hosts, thus becoming a major subject for biological research in the past two decades [4–6].

The hosted microbiota coexists with human cells in very different niches, ranging from the vast surface of the skin, the populous gastrointestinal tract and even within blood vessels and organs of healthy individuals [7, 8]. Viruses also inhabit places that have long been thought to be sterile [9], such as the urogenital tract and maternal milk [10]. Resident microbiota normally poses no harm to its animal host as the majority of its microbes form a symbiotic relationship with it, frequently as commensals and rarely as parasites [11]. Microbial profile configurations vary extensively between habitats, consisting of general and niche-specific types with varying abundances of cellular microbes including eukaryotic parasites, unicellular fungi, and a wide array of prokaryotes which comprise the majority of cells encompassed by the microbiota. Recent revisions to the estimates of the total number of prokaryotic cells inhabiting the reference male human (20-30 years of age; 70 kg; 170 cm) place it at over 3.8 × 10^13^ as opposed to a total of 3.0 ×v10^13^ estimated human cells, although the former represent ~0.3% (0.2 kg) of the total human biomass [12]. Yet, just the bacterial fraction of the human gut microbiota contains over 3.3 million different bacterial genes in its repertoire [7], exceeding that of the human genome by some 150-fold, which stands at ~19,000 genes over its 3.2 Mbp length [13].

Still, prokaryotes are far from being the most abundant representatives of the human microbiota seeing that the whole cellular fraction is susceptible to infections by an even larger number of specialized predators: viruses. As a group, these can potentially affect any type of cellular organism, ranging from human to bacteria, and together they comprise the human virome, which refers to the viral fraction of the microbiome [14]. The Latin term ‘virus' stands for ‘venom' or ‘poisonous fluid', and throughout history viruses were considered as something causing disease. In 1957, Nobel laurate André Lwoff provided the first exhaustive definition of viruses as separate entities, not as organisms or inanimate molecules [15]. Formally, viruses are considered potentially pathogenic obligate intracellular parasites with an infectious phase, devoid of a proper metabolism, which contain protected DNA or RNA molecules capable of replicating their nucleic acids and synthesizing viral components by hijacking the cellular systems of the infected cell. Eventually, they assemble new independent viral particles (virions) that are released after bursting the host cell, effectively starting a new infectious cycle (lytic cycle) or, in contrast, as temperate viruses integrating their DNA into the genome of the host or as a plasmid, rather than killing the host directly (lysogenic cycle) [15, 16]. Consequently, it has been hypothesized that some bacteriophages can modify bacterial communities and this in turn could affect dysbiosis. But, in spite of the parasitic nature of viruses, the human virome rarely presents any critical threat to the human organism as the great majority of such viruses target bacteria as their effective hosts (thus known as bacteriophages or phages for short) whereas systemic infections by eukaryotic viruses generally occur infrequently or, more commonly, as isolated events in healthy subjects [17].

At the global scale, viruses have a significant impact on ecology and evolution. They are the most abundant type of replicative entity on the planet (most of them are actually marine bacteriophages, containing 94% of all nucleic acid contents in the oceans), with conservative estimates reporting the existence of over 10^31^ concurrent virions at any given time worldwide, ten times the number of total prokaryotes which stands between 9.2 × 10^29^ and 31.7 10^29^ cells [18–20]. Together, they achieve over 10^24^ productive infections per second in prokaryotic cells, effectively wiping out 20-40% of the global prokaryote life daily and releasing their nutrients and CO_2_ to the environment [16, 21]. Furthermore, their role as genetic engineers is reflected in the 10^28^ bp of DNA that are transduced (viral-mediated transferred) each year by phages alone, overall contributing to horizontal gene transfer (HGT) across prokaryotes, although the aggregated number of different protein clusters in all viruses is estimated to be small, standing at around 3.9 million [14, 22].

In any niche, the virome is tightly associated to the prokaryotic fraction of the microbiota, physically sharing a common habitat and, in turn, contributing towards the modulation of the ecosystem by directly preying on its different components [23], as well as by moving genetic elements such as virulence factors (e.g. effector proteins for invasion, evasion of the immune system, phospholipases, proteinases, DNases, superantigens, adhesion factors, or mitogenic factors) [24–26] and antibiotic resistance genes (e.g. DNA synthesis and cell-wall-synthesis inhibitors, as well as genes coding quinolone efflux pumps) [27, 28]. Historically, it has been assumed from most culture-dependent studies, that phages have a rather narrow host range, limited to sole species or strains [18]. However, as protocols for multiple host-isolation have improved, it has become clear that in particular cases phage-mediated genetic exchanges can transcend the species and even the genus barrier as more viruses are now known to have a broader host range, spanning different bacterial taxonomic groups [29]. As phage research ventures deeper into the extensive terrain of the virome in the ‘omics' era, novel bioinformatic methods shed a new light into a more complex layer of viral-bacterial interactions [30].

## FROM THE AGE OF DISCOVERY TO AN ERA OF META-‘OMICS’

Viromics is a relatively new and burgeoning field of research undertaking to explore the virome (understood as the whole spectrum of viruses) within a specific niche, its ever-changing genetic component, the ecological and evolutionary impacts caused by the predation of cellular organisms by viruses, the resulting response of the infected cells, as well as the mobilization of genes affecting the fitness and survival of the viruses and their hosts. Yet, a significant fraction of the virome remains largely uncharacterized due to existing limitations in current methods and technology, which have historically relied on culture and microscopy techniques [31–33]. Virus research, however, has often been in the spotlight of scientific innovation, especially around the mid-20^th^ century, spearheaded by the Phage group (a notable network of brilliant scientists led by Max Delbrück) establishing the basis of molecular biology, as well as much of the fundamentals of cell biology and biochemistry, ushering the advance of genetic engineering, sequencing, and contributing to all biological sciences [34, 35]. As a field in its infancy, viromics is the latest example of the impressive adaptability of virology and will undoubtedly continue to develop during the following decades, broadening a much-needed perspective of the immense variability of viruses within complex ecosystems.

Similarly, the 20^th^ century saw the rise of virology, greatly owing to viral culturing techniques developed in the first half of the century, many of which remain relevant to this day. Viruses were to be considered as a separate type of entity in the last decade of the 19^th^ century, when a yet unseen Tobacco Mosaic virus (TMV) was independently confirmed by Dimitri Ivanovsky and Martinus Willem Beijerink (often considered the father of virology) to be transferable between tobacco plants in the form of bacteria-free filtrates [36]. Direct transmission between subjects by the inoculation of filtrates had been commonly used to study viruses. This changed when Frederick Twort discovered bacteriophages in 1915, demonstrating that viral species could be grown in cultures forming plaques of lysed cells on the bacterial lawn [37]. An equally important breakthrough would arrive in 1949 for eukaryotic viruses, after Franklin Enders, Thomas Weller and Frederick Robbins managed to grow isolates of polioviruses using various human embryonic tissue cultures [38]. In these, the cytopathic effect of viruses on cell cultures, evidenced by the formation of syncytia, lysis, detachment, or inclusion bodies, confirms viral presence, enabling the harvesting of viral particles of interest through filtration or gradient centrifugation techniques [39]. From this moment on, viral cultures became a major staple in the emerging field of virology as they would allow viruses to be reproduced safely and in sufficient quantities to study, providing the basis for serological assays, as well as for morphologic and molecular characterizations assisted by electronic microscopy, in the years to follow [40].

The development of sequencing technologies during the second half of the 20^th^ century ignited yet another lively period for virology, starting in 1972, when the first complete genome, that of RNA bacteriophage MS2 (a 3.5 kbp single-stranded genome encoding four genes) was published by the group of Walter Fiers, achieved with a radiolabeled 2-D fractionation method [41]. The first complete DNA genome would follow in 1977, when the group of Frederick Sanger, who had pioneered sequencing protocols in the mid-60s, published the complete genomic sequence of phage ϕX174 (a ~5.3 kbp circular single-stranded genome encoding 11 genes) as read from polyacrylamide gels using radiolabeled nucleotides and a ‘plus and minus' sequencing approach [42]. Sanger's would become the standard sequencing method for DNA genes and genomes after the introduction of dideoxy chain-terminators in the same year [43]. The procedure would gradually be automatized over the next decades by replacing radioactive labeling with fluorometric-based detection and shifting to capillary electrophoresis, coupled with robotized modules as well as faster and reliable computer-assisted detection of the fluorescent signals, eventually allowing hundreds of sequences to be analyzed simultaneously [44–47].

Sequence awareness changed the scientific understanding of biology profoundly, starting with the introduction of DNA-based molecular phylogenetics and its first glimpse of the tripartite division of life published by Carl Woese and George Fox in 1977 [48]. This transgressive new classification was based on the genetic variation of highly-conserved rRNA gene sequences (rRNA profiling), effectively separating the domain Archaea (originally Archaeabacteria) from the earlier Eukarya-Prokarya taxonomic dichotomy established by Roger Stainer and Cornelius van Neil in 1962 [49]. More importantly, Woese's group showed, for the first time, that all cellular life was related phylogenetically, with all lineages coinciding in a singular feature: ribosomes [48]. Techniques for rRNA profiling can provide an approximate evaluation of the taxonomic divergence between different related organisms (e.g. 97% identity is commonly regarded as belonging to a single species whereas 95% identity is used for genus) [50]. These were widely adopted in the 1980s, acknowledging their universality and their independence from culturing techniques, thus becoming the gold standard for prokaryotic taxonomic classification and replacing several morphology and biochemical approaches [51]. Contrastingly, DNA viruses lack genes encoding ribosomal proteins and rRNA or for that matter, due to their polyphyletic nature (they do not share a common ancestor), any type of strictly universal marker common to all viruses (RNA viruses have a RNA-dependent RNA polymerase) [52]. Even though some viral genomes actually carry sequences that are homologous to those found in the genomes of cellular organisms, viruses cannot be included in a single topology alongside ribosome-coding organisms but must instead be considered as a separate, yet ubiquitous, type of capsid-coding entities with a complex evolutionary history that is parallel to that of all three domains of cellular life [53]. Notwithstanding this, the advent of sequencing technologies would also bring a much required update to viral classification methods, which had historically been morphology-based or host-dependent [34].

By the end of the 20^th^ century, new scientific advances managed to overcome the need to culture microbes, a crippling constraint that had become a major challenge for microbial ecology. Recombinant DNA techniques developed during the early 70s allowed the cloning of target DNA by inserting it into a plasmid or viral vector, then loading it into culturable bacteria for copying, and finally harvesting the cloned inserts [54, 55]. The resulting high concentrations of pure DNA species were particularly suitable for the assessment of genetic markers such as rRNA profiling [56], the development of DNA probes for research and diagnostics [57], and eventually, for whole genome sequencing using a shotgun approach (consisting on fragmenting, cloning and sequencing a genome, then assembling the sequences together in a procedural manner) [58]. In 1986, Kary Mullis and collaborators published the method for polymerase chain reaction (PCR), consisting on the exponential amplification of fragments of target DNA using flanking primers [59]. At the time, enzymatic and immunologic assays were the only reliable culture-independent diagnostic tools for viruses [60] but PCR proved to be a totally revolutionary procedure, enabling the study of samples having very low concentrations of DNA (the lower detection limit varies among species; for example, ten copies of Influenza A virus can be successfully amplified for detection, given the right protocol) [61]. This paved the way, in 1991, for Stephen Giovannoni and collaborators to publish a novel type non-culturable sequencing experiment in which they managed to amplify pelagic bacterial DNA from samples from the Sargasso Sea using PCR amplifications targeted at the 16S rDNA of twelve randomly selected organisms [62]. Their results supported the hypothesis that most microbes in any environment are actually non-culturable by standard methods; as they reported, the SAR11 cluster from their dataset was comprised by a new type of bacteria, now classified as Pelagibacterales, formed exclusively by a non-culturable group of small, carbon-oxidizing bacteria that comprise around 25% of all plankton [63]. The metagenomic era had finally arrived and the idea of most habitats being sterile or populated by only a few microorganisms was discarded. Clinical practice has seen the largest impact since viral culture for diagnostics replaced in most clinical laboratories with PCR-based molecular assays [64].

## THE AGE OF NON-CULTURABLE ENTITIES

Crucial developments in molecular biology and genomics accumulating on the brink of the new millennium had enabled the scientific community to explore a larger picture of the microcosmos by ultimately obviating the need to culture microbes, kickstarting an era of systematic exploration of the unculturable fraction of the microbiota, led by first-generation sequencing technologies. Metagenomics was the term coined by pioneering author Jo Handelsman and collaborators in 1998 (her group was working with bacteria from soil samples at the time) to describe the study of genetic sequences obtained from “environmental” samples (that is, from the microbiota) using non-culturable techniques (hence the term meaning “beyond-genomics”) [65]. The foundations of the emerging field were set throughout the decade by groups working in habitats as diverse as the pelagic region of Sargaso Sea, hot springs in Yellowstone National Park, industrial-contaminated sediment from Seattle, human fecal samples, and soil, using 16S profiling techniques, basically undertaking general extraction from environmental samples, randomly amplifying molecules with PCR or cloning targeted at the 16S rRNA, Sanger-sequencing, followed by analysis [4, 62, 66–68].

At the turn of the millennium, the first steps had been taken towards integrating our understanding of the microbiota; however, the complete metagenomic catalogue far exceeded the information contained in a single genetic marker (such as the bacterial 16S rRNA gene) and the key to unveiling the whole metagenomic compendium would be found in the viral fraction of the microbiota. In 2002, the group of microbial ecologist Forest Rohwer published the first whole genome sequence (WGS) metagenomic survey of uncultured communities, also the first DNA virome, in two samples from surface water filtrates; this was achieved by the adaptation of random shotgun sequencing methods using cloned sequences from the viral metagenome [69]. The group obtained a 873 Mbp clone library and managed to assemble the resulting sequencing reads (henceforth reads) into contigs (longer sequences formed by assembling smaller reads), determining the assembly parameters from *in silico* shotgun simulations. Most viral genomes are shorter than those from prokaryotes, which simplifies the assembly. Yet, by comparing their sequences to those in the GenBank database, the group found that over 65% of all sequences found no homologs (‘hits' with database sequences) at the time, suggesting that much of the viral diversity was still uncharacterized. Furthermore, 57% of the phage hits were similar to genes with unknown function. This lack of information reflects the major limitation in viromics, a problem that continues to be addressed to this day. Despite the usage of filtrates and gradient centrifugation, the group reported hits with homologs in the Archaeal, Bacteria and Eukarya domains, and mobile elements comprise the majority of the identified contigs, another critical challenge that is unfortunately common in this field. Regarding viruses, and due to the experimental design, only DNA viruses were obtained and most were marine phages, including several that had not been sequenced before.

It should be noted that viromics has always been heavily reliant on DNA sequencing and consequently, most environmental assays had been focused on the DNA fraction of viromes, neglecting RNA viruses, a substantial fraction of the viral spectrum. The discovery of Hepatitis C by Choo and collaborators in 1989, was major proof of concept for the usage of unculturable methods and phylogenetics to capture and characterize novel viruses where there is no prior knowledge of the virus, the viral genome, and the presence of circulating viral antibodies [70]. The virus was found by screening a cDNA library obtained by a reverse transcription polymerase chain reaction (RT-PCR) with random primers using sequence hybridization. As the authors noted, the main challenge had been the insufficient quantity of viral nucleic acids present, along with a high level of host genomic DNA. In 2001, in an attempt to develop an experimental alternative to hybridization and immunological methods for analyzing viruses in commercial bovine serum, Tobias Allander and collaborators published their results of a survey they made of DNA and RNA viruses [71]. For the RNA fraction, the group adapted a 1991 protocol by Reyes and Kim for sequence independent, single-primer amplification (SISPA) to a general extraction of nucleic acids from bovine sera filtrated and then treated with DNases. In this procedure, cDNA libraries are created from RNA and special adaptors are ligated as primers for a PCR-like amplification, accomplishing the random enrichment of RNA [72]. The protocol allows full RNA genomes to be sequenced similarly to shotgun sequencing and is still in use today with minor changes. Allander's group managed to identify two new parvovirus species in their RNA dataset as common contaminants of commercial sera [71]. In 2003, using a sequence-directed metagenomic approach, Alexander Culley and collaborators published a culture-independent analysis of viruses to picornaviruses and related viruses in marine samples [73]. By designing primers for the RNA-dependent RNA polymerase gene from alignments of available picornavirus sequences, a sequence directed RT-PCR was carried out on the environmental samples. The resulting sequences were used to identify new viral families similar to picornaviruses.

Viromic assays had proven WGS metagenomics to be far more complex than 16S profiling but efforts continued nonetheless, facing new challenges to explore new domains and habitats. In 2003, Rohwer and collaborators published the results of the first study of the human DNA virome, taken from the fecal sample of a healthy 33-year-old individual [74]. Using a similar approach (vector-cloning, WGS shotgun Sanger sequencing), they analyzed the intestinal communities, which were reported to be enriched in phages but, despite the filters and gradients used to separate viruses, their datasets were mostly populated by sequences homologous to bacteria, something commonly affecting viromes in bacteria-rich habitats, regardless of the protocol [33, 75]. Most sequences identified as viruses were reported to be homologous to *Siphoviridae* and prophages (lysogenic phage genomes inserted in bacterial genomes), the latter being presumably integrated into bacterial genomes and a previously unaccounted complication in the analysis that blurred the line dividing prokaryotic and viral groups. The first two human RNA viromes were published independently in 2005, one by Allander and collaborators in the Netherlands [76], and the other by the group of Patrick Woo in China [77]. Both groups used the DNase-SISPA protocol proposed by Allander and collaborators in 2001 [71] with nasopharyngeal aspirate samples from patients respiratory infections, resulting in the identification of a new coronavirus. A larger study in 2006 by Zhang and collaborators, focused on the analysis of 18 fecal samples from healthy subjects from America and Asia. The eukaryotic viral fraction in their viromes was mostly populated by plant-infecting RNA viruses such as TMV and Pepper mild mottle virus (PMMV), an effect they attributed to diet and smoking habits, as confirmed by further studies [78, 79]. The virome, however, does not appear to become established by transient genera present in food as individuals following equivalent diets do not acquire a similar viromic configuration [80].

The first insights into prokaryotic WGS metagenomes would follow shortly, with Gene Tyson and collaborators managing to obtain near-complete prokaryotic genomes taken from samples of acid mine drainage biofilms thanks to the usage of larger metagenomic sets (over 70 Mbp) [81]. Higher-throughput data were clearly required as microbial genomes were larger than the previously explored viral ones. Also, metagenomics has an added difficulty compared to regular genomics: uneven sequence distribution among microbial species complicates the assembly. This is mainly because different genomes have a dissimilar copy number in the samples (depending mostly on the species' prevalence in the habitat and pre-sequencing methodological limitations) as well as polymorphic regions, further complicating classification. Thus, in order to separate reads into their respective genomes, researchers are required to assemble composite genomes considering the heterogeneity of each species while trying to avoid cross-species chimerism, a daunting task requiring high sequence coverage (number of copies sequenced, also referred to as the depth of sequencing) to ensure successful assembly. In the same year, Craig Venter's group raised the stakes by cloning a larger metagenomic library, obtaining an unprecedented total of 1.36 Gbp from surface water samples from the Sargasso Sea for Sanger sequencing [82]. About 25% of the reads in the dataset were successfully assembled into contigs, most of which belonged to genomes of the most abundant bacterial species but they also recovered double-stranded bacteriophages, especially in the singletons (unassembled reads appearing once).

Halfway through the first decade of the 21^st^ century, a new batch of high-throughput sequencing techniques gave rise to the second generation of automated sequencing platforms (next-gen platforms), ultimately democratizing metagenomics. Until then, the forbidding complexity of producing metagenomic clone libraries and the prohibitive costs of Sanger sequencing for whole libraries had made the adoption of metagenomics a rather slow process. Even though automated sequencing platforms produced large reads (700-900bp) early in the 2000s, sequencing was carried out at a very slow pace (< 80Kbp per day) [83]. The next generation of platforms was characterized by the real-time record of nucleotides being incorporated by polymerases, carried out in parallel using high-density multiple amplicon clusters. Besides, they required lower quantities of DNA for sequencing, in many cases obviating the need to clone DNA in vectors. They also significantly improved the total sequence yield, thus providing a cost and time-effective alternative to older approaches. The first of these was a sequencing-by-synthesis approach called pyrosequencing, commercially available in the form of the 454 Genome Sequence platform as of 2005 [84]. By coupling the light-emitting reactions of inorganic pyrophosphate synthesis pioneered in the 80s by Pål Nyrén [85] with a technique to carry out compartmentalized PCR within isolated water-in-oil droplets (emulsion PCR) [86], more than a million DNA molecules could be sequenced in parallel in less than a day.

The adoption of high-throughput datasets during the rest of the 2000s brought a much-needed expansion to the rRNA and viral databases, mainly provided by sequentially larger projects. In 2006, Edwards and collaborators (from the Rohwer lab) published a novel high-throughput sequencing study reporting the metagenomic analysis of natural mine water and sediment populations taken from over 700 m below ground in Minnesota [87]. They carried out 16S profiling as well using traditional cloning techniques for taxonomical analyses, and procured over 70 Mbp worth of metagenomic sequences for functional profiling after carrying out a whole genome amplification (WGA) approach using a rolling circle amplification protocol, a technique that randomly amplifies genomic DNA but applied to metagenomes [88]. They determined that metagenomes and 16S profiles were congruent, albeit the proportions varied, and that in metagenomes about 1 in every 10^5^ bases matched a 16S rRNA gene. Also, they calculated pyrosequencing was up to 30 times less expensive than with Sanger sequencing, although sequences were short and difficult to assemble and required WGA to generate sufficient DNA for sequencing. The output of 454 platforms would improve from 100 nt reads with 100 Mbp runs in 2006 to 700-800 nt reads with 700 Mbp in the 454 GS-FLX in 2016, when they were discontinued [89]. In the same year, the group of Angly and collaborators from the Rohwer lab published the results of the first large-scale environmental metagenomics survey, carried on coastal water samples from four oceanic regions in North America [90]. By analysing 184 viral WGS assemblies in 181 Mbp of pyrosequencing data, they managed to find core species distributed among the different samples, as well as species endemic to certain habitats. Most importantly, they helped expand the databases with sequences from the previously overlooked ssDNA viruses group. A few years later Craig Venter's ocean expedition would result in the publication of a massive 6.3 Gbp metagenomic dataset [91]. Surprisingly, this large project managed to surpass the yield of pyrosequencing high-throughput data by sheer brute force, resorting instead to older clone library and Sanger sequencing approaches. Nevertheless, it added an important collection of new viruses, mainly bacteriophages to extant databases.

Following the success of the 454 platform, different sequencing approaches were to join the second generation of sequencers. In 2006, Solexa, a startup founded in 1998 by Cambridge chemists Shankar Balasubramanian and David Klenerman, started commercializing the 1G Genome Analysis System (1G GA), capable of producing a 1 Gbp output [92]. Illumina acquired Solexa on the following year, releasing the GAIIx platform. The Illumina/Solexa approach depends on the simultaneous localized cluster-amplification of millions of DNA templates bound to a glass surface, followed by real-time sequencing by the detection of fluorescent emissions produced by the cyclic incorporation of labelled dNTPs acting as reversible terminators, optionally sequencing both strands of a fragment in Paired-End sequencing mode (the two overlapping reads can be joined bioinformatically to form a larger sequence) [93]. The adoption of Illumina platforms for metagenomics was initially slow because earlier iterations of the platforms were only suitable for genomic resequencing due to their short read output (between 30 and 50 bp for the 1G GA) when compared to pyrosequencing [94]. Eventually, Illumina would offer improved lengths with their Paired-End sequencing (2x150 to 2x300 bp) as well as specialized platforms including benchtop platforms with higher outputs enabling deep sequencing of the metagenome (1.2-120 Gbp) and production scale sequencers (1.5-6 Tbp), ultimately leading to the decline of the 454 platform in 2016 and granting Illumina the largest market share [89, 95, 96]. The last second-generation technology worth mentioning in the field is the hydrogen ions semiconductor sequencing, an alternative synthesis approach presented by Life Sciences (currently owned by Thermo Fisher Scientific) in 2010, in the form of the Ion Torrent platform [97]. With this approach, emPCR is carried out on target DNA and beads are held in microwells. Unmodified dNTPs enter sequentially, one at a time; ion sensors then record small fluctuations in pH resulting from the biochemical reaction of adding new nucleotides, allowing for up to 400 nt reads. Collectively, the second generation of sequencing platforms helped establish metagenomics as the burgeoning field it is today and is steadily extending to clinical practice [64]. Since 2015, a third generation of sequencers has shifted towards single-molecule platforms represented by Oxford Nanopore Technologies' nanopore sensing sequencing (MinION, PromethION) and Pacific Biosciences' Single Molecule, Real-Time (PacBio RS II and Sequel) platforms; however, their worth for metagenomics has yet to be fully demonstrated as they have mostly been used as complimentary methods for scafolding [98, 99]. Due to the limitations inherent to their particular technical approaches, each of the next-generation sequencing platforms (second and third generation) has certain key differences when it comes to performance but all of them generate usable datasets and together, they provide the robust and reproducible benchmark for culture-independent explorations that has revolutionized life sciences [64, 100]

While metagenomics grew in importance and costs were lowered by the advent of second-generation sequencing platforms, new meta-omics emerged for analysing different facets of the microbiota, paving the way for new multi-layered analyses. Metaproteomics, first introduced in a 2004 study by Wilmes and Bond, focused on the extraction and purification of the entire proteome of sludge samples from a wastewater treatment plant [101]. They loaded all proteins for 2D polyacrylamide gel electrophoresis and selected spots with high expression for peptide sequencing with a mass spectrometer. This first effort demonstrated the feasibility of carrying out proteomics on a mixed community. Similarly, in 2005, Poretsky and collaborators carried a microbial metatranscriptomic assay on the marine and freshwater bacterioplankton communities by directly extracting RNA transcripts [102]. After removing rRNA, they treated the preparations with DNases and amplified by randomly primed RT-PCR to generate adequate cDNA libraries for sequencing in a process similar to RNA viromics [71]. About 37% of the dataset was classified as belonging to unclassified organisms [102]. As noted by the authors, taxonomic classification drawn from a metatranscriptome permitted the reconstruction of a screenshot depicting the active fraction of the microbiota in samples highlighting actively expressed genes. It is undeniable that the integration of each layer of information contributes valuable information to a comprehensive understanding of a habitat and its microbiota, ranging from the genetic component to the characterization of the products of the associated microbial communities (environmental metabolome or meta-metabolome) but it also adds to the complexity of the analysis [103], an issue concerning systems biology but normally omitting viromic data.

## PENDING CHALLENGES

**Figure 1:**
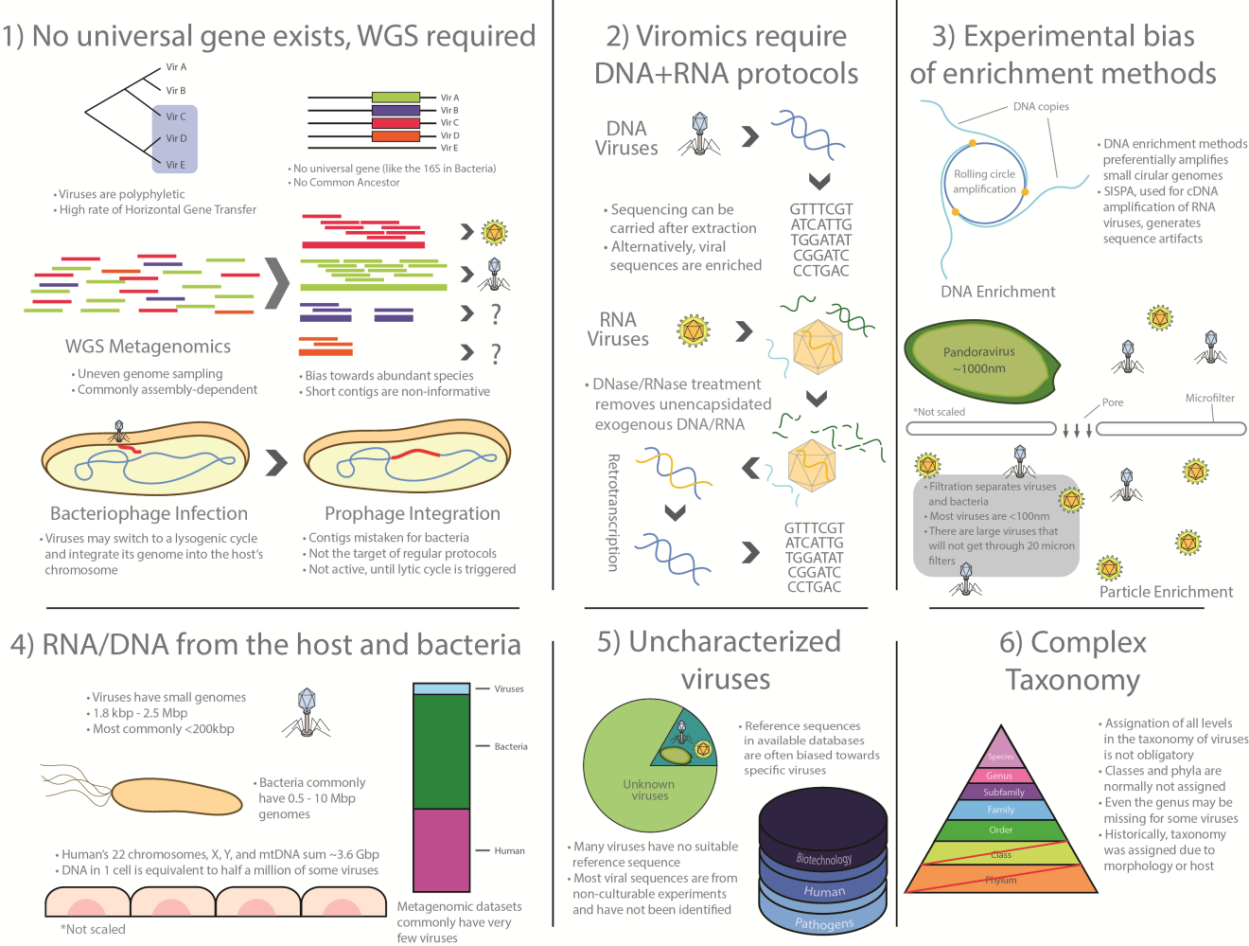
FIGURE 1: Challenges in the study of the virome. The study of viral communities faces numerous experimental limitations that are inherent to the study of viral particles. As viruses are polyphyletic, viromic-level phylogenies are often unreliable, further complicated by the lack of universal gene makers, a high rate of horizontal gene transfer, and the lack of a common ancestor. Viral metagenomics thus rely on the WGS framework which is commonly dependent of (yet, not restricted to) sequence assembly. This derives in additional challenges such as the formation of chimeric contigs, underrepresented fragments and species, and other general assignation issues. Additional hurdles are presented in viral RNA workflows as unencapsidated nucleic acids must be removed prior to RNA extraction, following retrotranscription. Additional biases may be introduced by enrichment of viral particles (filtration is shown as an example) or by amplification techniques for optional DNA enrichment, such as the rolling circle amplification. Niche ecology may also affect the recovery of viral sequences, as nucleic acids from the host and its bacteria are abundant in sample preparation and are often sequenced unintentionally. Moreover, suitable viral reference sequences are often missing from public sequence databases. Plus, several of those that exist lack a proper functional characterization or taxonomic assignation and databases are commonly biased towards specific species with pathogenic or biotechnological potential. Furthermore, current viral taxonomy is convoluted, with a large proportion of viruses having inaccurate or missing taxonomic labels, often derived from morphology or feature-based assignations. Together, these challenges have hindered the advance of viromics in the past decade, representing a hefty entry barrier for the scientific community due to the increased time, expertise, and resources that are required by these studies with respect to their bacterial counterpart.

In summary, viromics was the last successor in a long lineage of culture-independent approaches arising in the latter half of the 20^th^ century, and as such, inherited many of the advantages and the scope of metagenomic analyses. However, the field faces several unique methodological, ecological and conceptual challenges that represent key limitations to this day **(Figure 1)**:

1) No universal genetic marker (analogous to the rRNA in cellular life) is ubiquitous in the whole viral spectrum because viruses lack a structure derived from a common ancestor. Even though different markers such as capsids or polymerases have been used for the construction of phylogenetic trees [52], viromics relies on WGS methodology and thus inherits the corresponding challenges posed by large datasets produced by high-throughput sequencing. Particularly, WGS approaches produce uneven coverage variation that depends on technical and ecological factors. Although bioinformatics procedures have improved in the last decade, assembly is still cumbersome for species or strains having low prevalence in a niche, since most of them commonly have low coverage in a single high-throughput run, which translates into scarce and scattered fragments of their genomes [104]. This, in turn, produces complications in the downstream process related to data compilation into contingency tables in order to compare them ecologically, which is necessary because read distribution may not reflect actual species abundances.

2) Surveying the whole virome requires the use of two entirely different protocols for RNA and DNA viruses. Whereas the methods for processing DNA viruses fall closer to regular WGS methodology, RNA viruses comprise a significant fraction of the virome that is often ignored as it specifically requires retrotranscription of the RNA to cDNA, often using SISPA or similar protocols that complicate the downstream laboratory procedures and bioinformatic analyses. It is important to note that most limitations and issues inherent to metatranscriptomics also apply to viromic RNA workflows [105].

3) There is a marked bias towards specific groups of viruses due to experimental procedures. The most patent examples are possibly microfiltration, which retains large viruses, such as the ones infecting amoeba [8], and the multiple displacement amplification protocol (a WGA approach) used to enrich the often scarce viral DNA, as it works preferentially on circular ssDNA such as family *Anelloviridae* and in particular *Torque Teno Virus* which, coincidentally, have been reported as ubiquitous in humans by viromic analyses [106, 107].

4) Lingering RNA/DNA from the host (mainly in eukaryotic niches) and/or from the prokaryotic fraction are commonly reported, even after carrying out filtering, gradient separation, and RNase/DNase treatment of exogenous molecules. This translates into a reduced proportion of viral reads. As Alexander Greninger points out: a single contaminating host cell is the equivalent of half a million virions of some species (in total nucleic acid length) [105].

5) A large fraction of viral diversity remains uncharacterized and no reference sequences are available for most species. Although this problem continues to be addressed, viromics is still a largely unexplored field, resulting in the lack of adequate reference sequences for identification [104, 108]. Full characterization still requires viral particles to be isolated and cultured whenever possible, but most viruses are currently known only by their sequences. Viromic reads, however, are often classified based solely on their closest homolog, thus contributing to a less than optimal classification granting little information of the actual role of such viruses in the niche. Furthermore, a large proportion of the sequences that cannot be successfully identified in bacterial metagenomic sets are presumably part of the large dark matter of metagenomic studies, which may belong to viruses. Moreover, most taxonomic and functional assignations are currently being carried out using existing viral databases, most of which are biased towards a reduced group of pathogenic species or sequences with commercial interest for biotechnological applications.

6) Viral taxonomy is complex and a thorough revision is required to address consistency issues. Viruses were traditionally classified based on their morphology, serological testing or their host cells, without pertinent guidelines for nomenclature [109]. After the first viral sequences became available, it became clear that taxonomic groups were actually polyphyletic [110], further complicating taxonomic classification, which is in itself an imperfect system of organization. Viruses are classified into different taxonomic levels that are analogous to those in cellular life, the most common being order, family, subfamily, genus and species. However, taxonomic divisions in viruses do not always hold a biological or phylogenetic significance and several viruses lack classification at most taxonomic levels (orders and subfamilies are usually not assigned but even genera can be missing for some viruses). From a phylogenetic standpoint, structurally similar viral proteins can have varying evolutionary origins and, since viruses are not restricted to vertical transmission, ancestral lineages cannot always be traced or be determined by the hosts they infect, as these may vary as well [111]. This occurs because of the two-way HGT that occurs between some viruses and their potentially interchangeable cellular hosts (evidenced by the cell-derived metabolic and translation genes in viral genomes). Despite all these critical limitations, the field of viromics continues to expand.

## THE HUMAN HOLOBIONT

Even though viromic studies took a big step forward in the first decade of the 21^st^ century, it was actually microbial metagenomics that boomed after high-throughput sequencing technology became the standard for metagenomic explorations. The investigation of the human metagenome became a primary focus for biomedical sciences, resulting in the systematic exploration of the microbiota in human niches and, most importantly, the standardization of many protocols for general metagenomic research (both WGS and 16S approaches), in addition to the sequencing of novel strains to establish comprehensive reference databases. The gut metagenome, the most populated of all human niches, became the target of the European Metagenomics of the Human Intestinal Tract consortium (MetaHIT; 2008-2011), a 22 million € coordinated effort to establish the microbial composition associated to the habitat [112]. In 2010, the MetaHIT published the results of a multi-laboratory survey of stool samples from 124 European individuals sequenced using next-generation platforms, producing 576.7 Gb worth of sequences [7]. Over 3.3 million genes were reported to comprise the human metagenome, totaling over 150 times those in humans, 99% from bacteria. They also defined a core set of species common to most individuals, with Firmicutes and Bacteroidetes proving to be the dominant phyla. Moreover, they reported the prevalence of prophage related sequences (~5%) in the metagenome, suggesting bacteriophages play an important ecological role in microbial dynamics. In a follow-up study on European, Japanese and American populations, Arumugam and collaborators explored the functional diversity of the human gut microbiota, studying the distributions of clusters of orthologous groups (COGs) [113]. They reported that almost half of the metagenomic sets did not map to any COG. They also reported the detection of three species-driven groupings or enterotypes (*Bacterioides, Prevotella*, and *Ruminococcus*), not dictated by age, gender, body weight, or national divisions. These classifications have been strongly associated with long-term diets rich in protein and fat (*Bacteroides*) or fiber (*Prevotella*). In a similar type of analysis, the group of Brian Jones and collaborators defined four different putative virotypes within Bacteroidales-like bacteriophages within 139 human gut metagenomes [114]. These were associated to Bacteroidales from the Bacteroides and Prevotella enterotypes but were less well defined than Arumugam's and have been hypothesized to be extremes in an actually incomplete gradient.

Parallel to the MetaHIT, albeit having a larger scope, the Human Microbiome Project (HMP; 2007-2011) was established by the American National Institutes of Health to carry out the exploration of microbiota from diverse human niches, most notably, the gastrointestinal tract, oral cavity, respiratory tract, skin and vagina [115]. Whereas the MetaHIT had been heavier on functional profiles with a WGS approach, the HMP was centred around the diversity and dynamics of the microbial fraction of the microbiota (including longitudinal studies), having a strong 16S profiling and genome sequencing components using both 454 pyrosequencing and Illumina. The main results of the HMP consortium, published in 2012, included over 800 new reference strain genomes from all niches (most from bacteria but also including viruses and unicellular eukaryotes, ~5,000 16S profiles from 15 to 18 body sites in 242 healthy adults, most at three different timepoints in a two-year period, and 680 WGS sets from selected individuals [116]). Microbial species diversity was found to be highly variable, forming a seemingly continuous gradient among healthy subjects but showing strong niche compartmentalization, both within and between individuals [117]. Contrastingly, functional profiles and metabolic pathways showed a greater degree of conservation over time and among individuals, suggesting different species contributed to stable profiles, with core pathways including ribosome and translational machinery, nucleotide charging and ATP synthesis, and glycolysis. The gut and the oral cavity presented the highest microbial complexity (~400,000 families) whilst the vaginal niche proved the simplest (~16,000 families). Furthermore, the microbiota can change its functional and diversity profiles according to external stimuli, including the presence of foreign microbial species or viral infections and the occasional response of the host's immune system [118–120]. The WGS data produced by the HMP was further analysed by Kristine Wylei and collaborators in search for eukaryotic dsDNA viruses that could be sequenced as part of the libraries [121]. Viruses were found in 92% of the individuals, with an average of 5.5 viral genera per subject, including herpesviruses, papillomaviruses, polyomaviruses, adenoviruses, anelloviruses, parvoviruses, and circoviruses. Viromic profiles differed between individuals and some were conserved over time.

The human microbiota shows a complex nature of interconnected species displaying different dynamics, which are better understood as a system. In general, the human microbiota has been proven to be semi-conserved over long periods of time [117]. However, external stimuli such as the introduction of new species or the action of the host's immune system can alter its composition and abundance, as well as ecological drivers such as infection by local viruses (phages) or changes in nutritional intake [119, 120]. Interactions between the different groups comprising the microbiota are intricate and can have a significant ecological impact on the host. Different scenarios shape the microbial landscape, while nutrient availability promotes competition, synergetic efforts are also common, maximizing the exploitation by the microbiota, as well as cycles of colonization and biofilm formation [122]. The interactions of the different players in the microbiota have been mostly studied in the gut and they can be conceptualized as community-based ecological networks connected by syntrophic cross-feeding interactions (one species feeds on the product of another) [123]. In 2012, Kevin Foster and Thomas Bell reported net negative effects in experiments of mixed cultures, as most secreting species lack a beneficial effect, suggesting the dominant model is that of competitive interactions between members [124]. In the same year, a possible explanation for this phenomenon was proposed by Jeffrey Morris and collaborators, named the “Black Queen hypothesis”, based on a reductive genomic evolution driven by genetic drift. According to this premise, some functions can be considered dispensable due to metagenomic redundancy in comparative genomic studies [117] as several of them can be provided by neighbouring species. A contrasting hypothesis by Oliveira and collaborators points out that as selective pressure wanes and genes are lost, the metabolic interdependence established by complementing organisms and their reliance on molecules that may be intermittently available reduces the overall fitness of the system [125], though cooperation may be dominant when the gene pool is limited. On the same track, Coyte and collaborators reported cooperating networks were often unstable and that the host can exert immune suppression, spatial structuring and switching of nutrients to stabilize the system. According to their results, high diversity species may coexist in stable conditions when the system is dominated by competitive interactions because competition reduces the destabilizing effect of cooperation provided by co-dependence of its community members [119]. Regardless, the microbiota interacts directly with its host, as a healthy microbiota contributes to homeostasis by providing metabolic support via fermentation and degradation of nutrients [7], providing functional redundancy and modulating the immune response and signalling [126]. Recent insights into the gut-brain axis have also pinpointed microbiota as a major player in behavioural modulation in bidirectional communication with cognitive centres through immunological and neuro-endocrine systems associated with stress response, anxiety and memory [127]. Regarding viruses, some of the most important works have been carried out on monozygotic adult female twins and their mothers, the first by Alejandro Reyes and collaborators in 2010. They demonstrated intrapersonal variability of the virome was low and stable over time whereas interpersonal variation was high enough to differentiate between even twins, on the other hand, families shared a significantly similar distribution of viruses when compared to unrelated individuals; thus, no relation to the host genetics could be determined [75].

The momentum generated by the large human metagenome projects of the 2010s (most notably, the HMP and the MetaHIT) brought a renewed interest in human microbiota and its origins, which has been translated into countless publications. As a consequence, metagenomic studies have shifted from an era of exploration centred around modest 16S profiling to bold procedural WGS massive multi-layered systemic studies (few of which include viromics), in which genomes are sequenced and assembled by the thousand [152, 153], an impressive feature granted by the advance in sequencing technologies and analytical methods. More importantly, our perception of microbial communities has changed towards a more conciliatory view in which microbiota plays a pivotal role in the homeostasis of humans, forming a holobiont with its host. The concept of the holobiont (from Greek “hólos” for whole and biont for life element) was first proposed by Lynn Margulis in 1991 to describe [128] the supraorganism formed by the microbes and their animal host under symbiotic conditions. In fact, the study of microbial communities has demonstrated most of the resident microbes live not as parasites but as commensals or under interspecific cooperation [11] deeming microbiota a “forgotten human organ” due to its importance [129]. This revolution has also challenged the general perception of human niches, as in the case of the placenta, which was once conceived as a microbe-free pristine environment whilst humans were considered to be born sterile, acquiring their first microbes during delivery [130]. In 2008, Jiménez and collaborators challenged this idea in mice by orally inoculating labelled bacteria to the mother, managing to recover the same marked bacteria from the pups' meconium (the newborn's first stool, formed in the foetus before birth) and the amniotic fluid [131]. By the turn of the millennium, it had been confirmed that bacteria similar to that in the oral microbiome of mice were occasionally found, albeit in low numbers, in the umbilical cord and amniotic fluid in murine models [132]. In 2014, Aagaard and collaborators working in the HMP published an analysis of the first DNA molecules of bacterial origin found in human placenta and suggested their detection was not due to infection but to a mechanism by which mothers transfer bacteria to the foetus in their wombs [133]. The proposed microbiota of the placenta was nothing like the vaginal microbiota but rather displayed a striking similarity to that of the oral cavity so it was hypothesised to originate in the mothers' mouth by haematogenous (blood formation) spread, and delivered during early vascularisation and placentation. Subsequent studies detected bacterial DNA in the amniotic fluid and the meconium of humans [134, 135], leading to the hypothesis that there is an actual placental microbiota that provides the first exposure of the foetus to microbes, forming the basis of gut colonization and having potential lifelong implications for the training and establishment of the immune system [136]. In spite of these finding, the shift in the sterile paradigm has met fierce resistance as the scientific and the clinical communities remain cautious as to the details. Particularly, laboratory procedures, contamination of reaction agents have been criticised but, even if sequences truly belong to bacteria, they are present in very low quantities, which does not convince some of the existence of something as complex as a placental microbiome as they report these cannot be differentiated from background noise in controls or contamination [137, 138]. Regarding viruses, only pathogenic viruses are transmitted transplacentally or vaginally to the foetus such as, *human cytomegalovirus, human immunodeficiency virus, enterovirus, rubella virus, varicella-zoster virus, Zika virus*, papillomaviruses and influenza viruses; however, recent studies have failed to detect an actual virome in the amniotic fluid, nor detectable levels of eukaryotic viruses under normal conditions [138]. In recent years, however, studies of endogenous retroviruses in genomes have gained particular interest in placental biology as some of these vestigial retroviruses that have accumulated in the mammalian genome may be involved in cell–cell fusion and immune modulation in the placenta, although research has yet to establish the extent to which retroviruses have shaped the evolution of placental gene regulatory networks [139, 140].

Early dynamics in infants have been thoroughly explored to understand the maturation of the human microbiota, particularly that of the gut. It has been suggested that the method of delivery (vaginal or caesarean section) provides a differential colonization of microbes in the first weeks of life [141]. Whereas infants born vaginally have an initial microbial configuration that resembles that of the mother's vagina (rich in *Lactobacillus, Prevotella*, and *Sneathia spp*), infants born through caesarean section display a microbial configuration that is closer to the mother's skin microbiome (rich in *Staphylococcus, Corynebacterium*, and *Propionibacterium spp.)* [142]. In 2014, Jakobsson and collaborators reported that infants born from caesarean sections had reduced microbial diversity during the first two years of life whereas vaginal delivery favoured the maturation of the immune response through Th1-like responses [143]. Likewise, breast milk (previously considered to be sterile) may represent a viable source of microbes for mothers to pass their microbial configurations to infants [10, 144]. Bäckhed and collaborators, on the contrary, reported this difference is maintained for the first year of life alone [145]. By inheriting the mother's configuration, the pattern recognition receptors of the immune system of the infant is exposed to bacteria bearing microbe associated molecular patterns (now known to be not only exclusive to pathogens), therefore helping on the maturation of the system by preventing future inflammatory responses from arising, triggered by commensal species [146]. However, according to Chu and collaborators, differences in the microbiota between caesarean section and vaginal delivery neonates are not detected as significant six weeks post-delivery, after which they also show niche specialization [146]. After that, the infant microbiota remains highly variable, changing notoriously after the introduction of solid food, eventually stabilizing after approximately three years [147, 148]. Similarly, elderly people present a higher inter-individual variability in microbial composition when compared to young adults [149]. Contrary to the intrapersonal stability displayed in adults demonstrated by monozygotic twin studies [75], in 2015 Lim and collaborators reported that the virome is rather unstable in infants, generally dominated by bacteriophages but containing some eukaryotic viruses [150]. By studying the virome of infant twins, they reported a shift from a highly diverse community dominated by phages from the Caudobacteriales order in the first two months of life, to a domination by phages from the *Microviridae* family after two years of life occurring after an overall decrease in viral diversity, which also coincides with the moment the microbial configuration starts to resemble that in adults [148]. Contrastingly, Enteric eukaryotic viruses such as *Adenoviridae, Astroviridae, Anelloviridae, Caliciviridae, Picornaviridae*, and *Reoviridae* are reported to have limited persistence over the same period [150].

## CONNECTED WORLDS

As in other niches, phages are an abundant group in human niches but their dynamics is still a matter of debate. Early studies pointed towards a predatory kill-the-winner model in which viruses act as natural predators of bacteria, specializing on species that thrive and reducing their levels to normal [151]. Since the first human virome studies were published, Caudovirales phages were detected as part of the DNA landscape, represented by species from families *Siphoviridae, Podoviridae* and *Myoviridae* [74]. The decreased cost and ease of sequencing in the 2000s led to an important increase in the number of complete genome sequences available for different bacterial strains from the same species, resulting in the development of the pan-genome concept: as a result of intraspecies evolution, synteny is shared only in a core set of genes clusters shared across species, flanked by metagenomic islands of diversity that are transitory for the species; the pool containing the core (65-90%) and accessory genomes (10-35%) of a species is known as the pan-genome of the species [152, 153]. In environmental metagenomics, and contrary to culturing conditions, WGS procedures draw random fragments from the complete pan-genome of non-clonal strains present in a sample, it can be expected that genomes reconstructed by assembling short reads (e.g. the output of high-throughput sequencing) are in fact the reflection of the inner variability that exists within a species in the sample, with the most prevalent strains contributing the most towards the core genome. In general, the larger the read output is, the greater depth of sequencing (coverage) per species, resulting in a more complete pan-genome, which makes this a good target for single genome high-throughput sequencing and ultra-deep sequencing. This is not only relevant for assembly but for understanding the regulatory role that viruses might play in their ecosystem. In nature, bacteria must adapt, not just to available resources and to physical conditions, but they must also constantly compete against opposing agents that coevolve with them, such as bacteriophages (following the Red Queen hyphothesis: viral and bacterial species undergo antagonistic evolution to remain competitive and avoid extinction) [154]. From an evolutionary point of view, a possible mechanism for bacteria to fend off phages consists of having different versions of their extracellular proteins that are potential targets for phage receptors. Precisely, Rodriguez-Valera and collaborators reported genes coding for the O chain of the lipopolysaccharide, as well as exopolysaccharide biosynthesis clusters and genes involved in sugar modifications of extracellular structures are some of the most variable in metagenomic studies, part of the accessory genome of species, even in those species having an extremely compact genome [153]. Overall, variability is kept stable over time and these genes are also as overrepresented as the genes involved in nutrient transport and environmental sensing, which further supports the prevalence of kill-the-winner dynamics. The long-term maintenance of such variability in different strains allows a single species to maximize the exploitation of resources in the system and, predictively, prevents a single phage infection from wiping out the entire population since the occasional evolutionary advantage of a single variant is alleviated by bacteriophage action (the success of infection from a single viral lineage becomes increasingly probable as such an overfitted variant becomes fixated in the population, effectively redressing the balance in the strain population). In summary, a species pan-genome with a large accessory genome is indicative of constant variation in the strains and the maintenance of such long-term diversity suggests kill-the-winner dynamics [155]. Contrastingly, a small pan-genome is indicative of constrained populations with little phage interference such as biofilms resulting from a clonal sweep of strains with the fittest genome [156].

### CRISPRs and bacteriophages

Before taking over the genome-editing world, the CRISPR-cas systems were studied as bacterial elements that code for an adaptive immunity in prokaryotes against exogenous DNA of viral or plasmid origin, an important feature providing information about the virus-bacteria relation. In 1987, Yoshizumi Ishino and collaborators reported an accessory nucleotide sequence in *Escherichia coli*, consisting of five 29 nt repeats interspaced by unique 32 nt sequences [157]. Similar sequences with diverse lengths and number of repeats were discovered in different strains of *E coli* and other species of bacteria and eventually in archaea, where they turned out to be more prevalent; they all featured the same type of structure: short repeats interspaced with equally short unique sequences not sharing the same sequences (even completely different in phylogenetically-related strains) [158]. In the 2000s, they were recognized as mobile elements existing in prokaryotic genomes and plasmids and came to be known as clustered regularly interspaced short palindromic repeats (CRISPR), flanked by an upstream leader sequence and adjacent to CRISPR-associated genes (*cas*) [159]. The function of CRISPRs became apparent after Tang and collaborators detected the complete transcription of CRISPR genes in long RNA (pre-crRNA) that are subsequently edited into small RNA molecules (crRNA) bearing the length of a single spacer-repeat unit [160]. This transcription, directed by the leader sequence, works as a defence mechanism in prokaryotes. Each crRNA is derived from exogenous sequences of bacteriophage or plasmid origin and favours resistance to infection by phages carrying the sequences in the spacers, as described by Mojica and collaborators in 2005 [161]. New spacers are directly derived from bacteriophage or plasmid sequences introduced to the cell during past infections as a sort of immunological memory in prokaryotes [162] and at least one CRISPR locus was detected in over 40% of sequenced bacteria and most archaea [163]. The interference was experimentally demonstrated by the group of Barrangou in 2007, by exposing *Streptococcus thermophilus* colonies to infection by phages φ852 and φ2972 and subsequently detecting the corresponding phage/plasmid-derived spacers within the CRISPR sequences in strains surviving infection [164]. In general, the mechanism consists of direct DNA targeting (Type III systems can target RNA instead) by the crRNA followed by nuclease activity on the complementary infectious DNA (protospacer) produced by different Cas proteins (other Cas proteins participate in the cleavage of exogenous DNA for the incorporation of new spacers, the cleavage of pre-crRNA and the formation of the antiviral complex) [165]. More importantly, the precise mechanisms vary among the three known types of CRISPR systems (Type I, II and III, using Cas3, Cas9 and Cas10, respectively) but in all of them, CRISPR loci can be used to access a historical record of viral infections linked to a specific prokaryotic strain [166].

Under natural conditions, prokaryotes and viruses interact in highly complex scenarios presenting markedly different evolutionary dynamics. In 2008, Kunin and collaborators studied strain variability of *Candidatus* Acummulibacter phosphatis, an unculturable species comprising up to 80% of the biomass in Enhanced Biological Phosphorus Removal sludge communities in an effort to describe the evolutionary dynamics and the role of bacteriophages [167]. They detected a highly conserved pan-genome in two geographically distant populations, where the accessory genome had highly variable sequences coding for extracellular polymeric substances (a first line of defence against phages that masks potentially exposed receptors) and five main CRISPR, presumably resulting from recent evolutionary dynamics to counter phages. CRISPR sequences contained different spacers between the two populations, and it was demonstrated that viral sequences obtained from the same habitat matched the spacer sequences, with some spacers targeting more than one related phage. They hypothesized that the high degree of identity between the two populations and the highly local variability of genomic items for defence against phages was the result of kill-the-winner dynamics. As mentioned before, in 2010, Reyes and collaborators published the analysis of the virome, 16S and community metagenome of four pairs of adult female monozygotic twins and their mothers on three different timepoints, using high-throughput sequencing [75]. Of the total identifiable viral reads, 25% were reported as coming from bacteriophages and prophages, with most of them being classified as temperate viruses (at least potentially) infecting Firmicutes or Bacteria. Twin-mother groups presented a significantly similar virome, but each set was unique to each individual, and dissimilar bacterial profiles. Also, intrapersonal diversity in the virome was stable, as 90% of the most common viruses were retained over time. They also analysed over 2000 different CRISPR spacers from the metagenomic datasets that could not be related to the corresponding virus-like particles (VPL) in the viromes. In general, their results did not support the predatory viral-microbial dynamics, and the fact that they found high abundances of dominant phages with little divergence over time, as well as the widespread integrases in the viromes, provided the bases for the hypothesis that temperate phages might play a larger role in the gut ecosystem. In this scenario, low-energy conditions in the gut may induce prophage activation, something that Reyes and collaborators verified by inoculating gnotobiotic mice with two sets of bacteria containing two or three temperate phages respectively [75]. By measuring expression of viral markers, they detected that most prophages remained in a lysogenic cycle while one of them clearly became activated in the gut (but not *in vitro* with several tested carbon sources).

The paradigm of viruses negatively impacting microbial populations has been challenged in the last years as the result of expanding metagenomic studies has proven prophages are widely distributed among prokaryotes [168]. A new alternative ecological conception proposes that infection by viruses may confer an advantage to both phages and prokaryotes under specific circumstances. Cyclic oscillations in the numbers of dominant bacteria in environmental niches have traditionally been modelled by Lotka-Volterra equations for predator-prey systems [169] but these conditions occur most commonly in habitats with rich nutrient concentrations. In 2016, Knowles and collaborators determined that virus-like particles are relatively less abundant in habitats presenting high microbial abundance, resulting from restricted lysis, as experimentally detected in coral reef samples and tested *in silico* in other habitats including human [170]. As an alternative to the kill-the-winner model, they proposed the piggyback-the-winner model in which lysogeny is favoured in high host density conditions supported by increased representation of integrase and excisionase genes in the virome. The rationale behind the model is based on the observation that generalist “nested” phages infecting a bacterium confer them a resistance to further infections by other phages, thus superimposed infections (superinfection), a process that is preferable as it lowers the cost of generating resistance for the bacteria and of disseminating for the virus [171, 172]. Also, HGT resulting from lysogeny may provide an adaptative advantage [170]. Both models seem to operate under different conditions but it has yet to be determined what causes them to switch in complex communities. As Barr and collaborators noted, dynamics seems to be more complicated in mucosa of animals, for example in the human gut and lungs, where viruses bind to glycan domains that coat mucins using Ig-like proteins exposed on their capsids, increasing the probability of collision with viable bacteria for infection [173]. As the authors suggested, the establishment of phages provides the host with a non-host-derived form of immunity against certain bacteria. About half of these viral communities were reported to be temperate, although lytic infections were reported to dominate. Silveira and Rohwer have recently proposed a model dependent on a special structure to reconciliate the kill-the-winner and piggyback-the-winner models in mucosal communities [174]: Based on the gradient concentration of mucin on epithelial surfaces, viral concentration is expected to be positively correlated (with higher levels closer to the epithelial layer), contrary to bacteria. Thus, lysogeny is favoured in the overpopulated top layers of mucus (physically distant from human cells) with several commensal bacteria hosting prophages in their genomes that confer them resistance to superinfection. When a bacterium gets closer to the inner layers of the mucus, where bacterial cell growth and density decrease, prophage induction is favoured and the phage enters a lytic cycle and bursts its host, replenishing the high-density area with free virions.

As part of the arms-race between viruses and prokaryotes, a viral mechanism to counter the change in the bacterial receptors involves mutating their receptor-binding tail fibres [175]. Recent related studies have drawn attention to diversity-generating retroelements (DGR), a type of sequence encoding for an error-prone reverse transcriptase originally reported in 2002 by the group of Jeffrey Miller in bacteriophages infecting Bordetella species [176]. It transpired that DGRs were being used by bacteriophages for directing mutagenesis through faulty adenine pairing to switch host tropism by selectively changing the sequence of their phage tail fibres [177]. Later, these sequences were confirmed to be scattered throughout phylogenetically distant archaeal and bacterial genomes as well as in viruses, including inhabitants of human niches, providing microbes with a rapid mechanism to respond to environmental changes through mass sequence diversification [178]. The scale of sequence variation has been recently paralleled to that in vertebrate adaptive immune systems by the group of Partho Ghosh [179]. In 2018, Benler and collaborators reported the survey of viral metagenomes in which they discovered 92 new DGR sequences exclusive to bacteriophages, most of them in prophages within Bacteroidetes, Proteobaceria and Firmicutes [180]. After the induction of one such phage in *Bacteroides dorei*, the authors demonstrated that it had and ample host range, suggesting DGR contributes to a ubiquitous mechanism in human niches for the interaction between viruses and bacteriophages, contrary to previous analyses of the gut viral metagenomes demonstrating few genotypes are shared among individuals (they estimated it to be present in about half the human population). Work on the DGR elements continues and are gaining momentum due to their biotechnological potential.

## VIRAL TAXONOMY AND DATABASES

The last few years have seen the resurgence of viromics. As sequencing platforms, now dominated by Illumina, have increased their total throughput and reduced the overall costs of WGS approaches, a much-needed expansion to viral metagenome diversification is now painting a bright future for this field. The advent of automated sequencing has brought an unprecedented increase in the rate at which metagenomic data is being generated, with worldwide capacity doubling almost every nine to twelve months over the past 20 years [181]. As a result, publicly available sequence databases keep growing exponentially, as has the difficulty in handling such volumes of data [182]. Data extrapolation by Stephens and collaborators in 2015 estimated the annual global sequencing capacity, which at the time of this writing stands in the petabasepairs range (1 petabasepairs = 10^15^ bases) and could reach ~1 Zbp (1 zettabasepairs = 10^21^ bases) by 2025, requiring 2-4 Ebp (1 exabasepairs = 10^18^ bases) for storage [183]. Paradoxically, the world capacity to analyze data does not cope well with such a scenario as the rate at which computational power increases cannot keep up. Recently, bioinformatic studies re-analyzing public metagenomic datasets have shown that, even now, we are generating more metagenomic data than we can reasonably analyze, perhaps best exemplified by the discovery of the ~97kbp genome of the *crAssphage virus* by the group of Dutilh and collaborators in 2014 [184]. This elusive non-culturable virus was detected by re-analyzing WGS data from Reyes *et al.* 2010 [75] consisting of the gut virome of four unrelated families formed by twin pairs and their mothers, which was found to be the most abundant Viral-like particle in the set (comprising 22-90% of all reads in the samples) [184]. Presumably, it had been ignored because predicted crAssphage proteins had no homologues in the databases at the time of discovery and further analyses with CRISPR sequences of co-occurring bacteria postulated it as a putative *Bacteroides* phage. An exhaustive exploration of crAssphages in the largest sequence repositories was carried out by the group of Yutin and collaborators and published in 2017, proving crAssphages belong to a widespread family regularly found in the gut, which presumably prey on bacteria from the Bacteroidetes phylum and, morphologically, would be classified in the Podoviridae family (from predicted tail proteins). Taxonomy is, however, one of the greatest challenges in viromics today.

Virus classification has been a matter of debate since the first systematic schemes were proposed back in the 1940s and has experienced a rough migration towards the sequence-based taxonomic era, especially after the introduction of non-culturable methods and high-throughput sequencing. In 1948, prominent plant virologist Francis Holmes presented a compilation of the 248 “filterable viruses” that were known at the time, complementing his previous work on plant viruses, as part of a supplement to the 6^th^ edition of the Bergey's manual of Determinative Bacteriology, the reference manual for nomenclature [185]. In Holmes' classification, viruses were assigned to the “groups whose relationships are obscure” with the order Virales and were given suborders according to their host (animal, plant or bacteria), and Latin Linnaean binomials based on the filter pore size, the type of disease or the type of tissue they infected. After the 1950 meeting of the Virus Subcommittee of the International Nomenclature Committee, expert virologist Christopher Andrewes (who had proved bacteriophages were viruses) fiercely criticized Holmes' classification for ignoring the properties of virus themselves and decided to drop binomial names in favour of the suffix –“virus”, which has remained for many eukaryotic viruses to date [186]. Virus had already been seen under the electron microscope so Andrewes suggested eight criteria for a new classification: morphology, chemical composition, immunological properties, susceptibility, transmission, host, pathology and symptomatology, specially emphasizing antigen detection due to his background work on influenza viruses. A decade later, microbiologist and Nobel laureate André Lwoff presented a proposal for a new classification at the symposium of Basic Mechanisms in Animal Virus Biology held in 1962. His nomenclature was based on the type of nucleic acid, which was gaining momentum at the time, the symmetry of the capsid, whether the capsid is naked or enveloped, and the number of capsomers [187]. Following a similar approach, molecular biologist and Nobel laureate David Baltimore proposed one of the classification schemes that is in use to this day, referred to as the “Baltimore classification” [188]. According to this method, viruses are assigned to groups I-VII (group VII was actually appended *a posteriori*, after the genomic dynamics of the Hepadnaviridae family was described [16]) according to the type of nucleic acid of the viral genome and the steps necessary to synthesize the mRNA molecules required for viral protein translation in the host [188], as follows: Group I, comprised of dsDNA viruses, produce mRNA directly. In Group II, ssDNA viruses must first create the complementary negative sense (-) DNA strand, then the mRNA. In Group III, dsRNA viruses can use negative (-) strand as template for the mRNA, as they do in Group V viruses of (-) ssRNA genomes. Positive stranded (+) ssRNA viruses in Group IV can either use their genome for translation or create a (-) RNA intermediary as a template for mRNA. Group VI contains retroviruses, (+) RNA viruses that are first retrotranscribed to (-) DNA, then transcribed to mRNA. Group VII was added later, based on the description of the *Hepatitis B virus* (HBV) [16]. It is characterized by viruses having a dsDNA-RNA+protein hybrid genome, where the DNA is interrupted by a short (-)RNA segment. The DNA fraction is completed into a full circular dsDNA molecule after the removal of the RNA and the protein, then the mRNA is transcribed from the + strand.

Despite all the isolated efforts to standardize nomenclature, little to no control over naming existed prior to the 70s, as it was customary to keep the name given to viruses by their discoverers, irrespective of any taxonomic convention. In order to address this, and considering the pace at which the field was growing at the time, representatives of the International Association of Microbiological Societies across the world were appointed to form the International Committee on Nomenclature of Viruses, founded in 1966 (currently the International Committee on Taxonomy of Viruses or ICTV) [189]. They were tasked with the laborious endeavour of consulting virologist worldwide with a view to proposing general guidelines for a universal system of viral nomenclature and taxonomic classification, independent of their hosts and bacteria naming conventions. They then voted on the approval of the proposed classifications, releasing periodic reports on the state of viral taxonomy. Their first report was presented in 1971 and included 290 approved and a similar number of candidate viruses [190]. Regarding the former, viruses were assigned to one of 43 “groups” (later genera), but only two families were recognized (for vertebrates). Also, there was no taxon equivalent to species as the concept of virus having species was controversial and had not been accepted yet. Ever since then, the ICTV members have gathered every few years to discuss the addition of proposed viruses and taxonomical categories, issuing a total of ten reports over the 52 years of its existence. Notwithstanding, at the outset the ICTV's authority and its methods were questioned especially by plant virologists, who argued against the species concept. This changed after the adoption of Marc van Regenmortel's definition of viral species: “a polythetic class of viruses constituting a replicating lineage and occupying a particular ecological niche” [191]. The inclusion of the species label by the ICTV drastically changed viral taxonomy as it became its central feature as seen in the 5^th^ report (1991) onwards, and resulted in a major restructuring of multiple families, the adoption of genus instead of groups, and the inclusion of the order and subfamily levels in the 6^th^ report (1995) [192]. The latest report (Oct 2018) introduced phyla and subphyla and recognizes the existence of a total of 4,958 species, 846 genera, 64 subfamilies, 143 families, 14 orders, 2 subphyla, and 1 phylum [193]. Apart from that, there is a large list of unidentified viruses pending classification.

As mentioned in a previous section, complexity of the current viral taxonomy is a crucial challenge that has yet to be addressed in virology and the one aspect that has been most affected by the advent of viromics. Although the ICTV has ultimately been accepted as the authority for the classification of new viruses with the adoption of their viral taxonomy, it has been not without serious criticism, most importantly, regarding its failure to address some of the biggest shortcomings in naming conventions and to adapt to the high-throughput era [108, 109, 194–196]. The following is a list of some of the most important limitations of the ICTV current guidelines (available online [197]): Current rules concerning assignation are only applied to species and lower levels (meaning ICTV is not responsible for the names of serotypes, genotypes, strains, variants and isolates) and species naming rules are somewhat lax. Although recommended, the use of all levels of the taxonomic hierarchy is not compulsory, and this often results in species lacking a genus or other lower level taxonomies. In fact, even though the ICTV has defined suffixes for taxonomic levels (“-viria”, “-vira”, “-virae”, “-virites”, “-viricota”, “-viricotina”, “-viricetes”, “-viricetidae”, “-virales”, “-virineae”, “-viridae”, “-virinae”, “-virus" and “-virus” for realm, subrealm, kingdom, subkingdom, phylum, subphylum, class, subclass, order, suborder, family, subfamily, genus and subgenus, respectively), only since 2018 have there been viruses applying more than the commonly used order, family, subfamily, genus and species. The validation process for new taxa or species takes time as the relevant subcommittees and study groups must be consulted. For this reason, the executive committee of the ICTV organizes annual meetings [192]. Furthermore, taxa is only accepted when representative members are well characterized, which is not commonly the case in metagenome datasets, although this was partially addressed in the 2016 meeting to facilitate the classification of unculturable virus sequences [190]. This decision has polarized ICTV groups as in some cases only the sequences are available for a virus without any additional information about the viruses themselves [198]. Finally, perhaps the greatest controversy facing the ICTV was the change in species definition in 2013: “A species is a monophyletic group of viruses whose properties can be distinguished from those of other species by multiple criteria” [197] which is not compatible with the current conception of a virus and the state of the viral taxonomy today. The rationale, however, is based on an attempt by the ICTV to redefine viral classification under a phylogenetic framework. The extent to which this is applicable is questionable due to HGT and the lack of a universal marker and it has met with resistance from the scientific community [198].

Another important limitation for viromics concerns the references in databases used to identify viromic sequences. Even though sequence databases have grown in step with advances in genomics, and more recently the introduction of high-throughput sequencing, they have mostly been biased towards pathogenic viruses, particularly those affecting humans and commercially relevant crops. Besides, biological data is accumulating at an unprecedented speed and dozens of new molecular biology databases appear each year. In fact, back in 1988, in an effort to organize the huge amounts of data flooding online repositories, arising from the popularization of sequencing techniques, the Los Alamos National Institute created the Listing of Molecular Biology Databases. This was the first thorough international directory of biological databases, which, incidentally, included only one viral database, concerning DNA and amino acid sequences from AIDS-related animal viruses [199]. With the popularization of online informatic resources, the list eventually derived into the Molecular Biology Database Collection (MBDC at http://www.oxfordjournals.org/nar/database/c/), a curated compilation of openly accessible online databases and their characteristics, accompanied by the annual publication of the Nucleic Acids Research database issue that reports new additions and deprecated entries, currently in its 25^th^ edition [200]. The list is growing steadily with each iteration, evidence of the fast pace at which biological sciences advance. Just in the last three years, 199 new databases have been accepted while only 100 obsolete ones were removed [200–202]. At the time of this writing, there were at least 40 different specific viral-related databases in the list as well as several non-specialized ones containing general viral sequences. Although most viral databases in the MBDC are not created for metagenomic-scale virus explorations in the line of viromics, they can prove useful nonetheless. The MBDC includes species-specific databases such as the HBVdb (for everything related to *Hepatitis B virus*), IVDB (for influenza viruses), or the HIV Drug Resistance Database (for *Human Immunodeficiency virus*), as well as pathology-related databases and of clinical interest such as the HFV database (for haemorrhagic fever viruses), ViPR (for viral pathogens in general) and AVPdb (experimentally validated antiviral Peptides). Some of the databases include information other than sequences such as the structural VIPERdb (icosahedral capsids), and the ViralZone (molecular and epidemiological information), or features derived from genomic analyses such as the pVOGs (prokaryotic virus orthologous groups), phiSITE (gene regulation in phages), PhEVER (phylogenetic and evolutionary relationships), MVP (viral-bacterial interactions), MRPrimerV (PCR primers for RNA viruses), and the ICTV taxonomy (included since 2018). General purpose sequence repositories such as the IMG and GeneBank, are also available. Even though the online search interface seems rudimentary and some links need to be updated, the list is a general reference in the viral study framework. Also, many of the databases provide analytical tools.

Nowadays, most freely available DNA and RNA sequences in specialized databases include curated data, obtained from what can be considered the most important biological data repositories, the collection coordinated by the International Nucleotide Sequence Database Collaboration (INSDC). There are three nodes to this global initiative, operating collaboratively since 1987: the DNA Data Bank of Japan (DDBJ) of the National Institute of Genetic in Japan, the European Nucleotide Archive (ENA) of the European Molecular Biology Laboratory's European Bioinformatics Institute (EMBL-EBI) in the UK, and the GenBank at the National Center for Biotechnology Information (NCBI) in the USA [203]. Sequences and annotations submitted to any of the three nodes are made readily available in the other two and can be accessed through each member's interface in mirrored repositories that are regularly updated, providing a consistent backup for archival preservation. In its latest report (August 2017), the INSDC databases amassed a shared total of 2.65 Tb (1 Terabase = 10^12^ nt) nucleotides from ~900 million sequences in its traditional archive of assembled annotated data, representing a 185% increase in two years. The INSDC also accepts raw sequence reads and alignments from high-throughput sequencing in its Short Read Archive (SRA) which surpassed the size of its traditional counterpart long ago, summing 3.2 Pb (1 Petabase = 10^15^ nt) worth of sequences in the last report (growing at a rate of 210%). The INSDC uses the an ICTV-based taxonomy for its viral sequences and includes the Baltimore classification (type of nucleic acid) as additional unranked data. However, viral annotations are limited (4149 species were reported in Aug 2017 [203]) and viromic sequences are commonly deposited as raw data in the SRA, thus they are rarely given reliable taxonomic information (due to the lack of homologs) and therefore remain uncharacterized. Although looking for viruses in the unannotated data of the INSDC can be a daunting task, many of the WGS metagenomes can prove useful for viromics.

## GOING FORWARD

As established by this review, there is still much work to do in terms of improving the framework for viromic studies and perhaps the answer lies in the generation of even larger databases and tools for big data analysis. There are plenty of online systems that provide automated tools for annotation and analysis of metagenomes, such as the EBI Metagenomics platform, MG-RAST and the IMG system service, which provide a user-friendly environment for high-throughput data processing [182, 204, 205]. Still, most of their servers are not intended for virome analyses and usually ignore sequences that bear no homology to microbial sequences in extant databases. This, however, as recently demonstrated, this has been a critical missed opportunity, since the massive collections of metagenomic data flowing through these platforms may bear precious information regarding viruses, as yet unanalysed. Indeed, in 2016, Paez-Espino and collaborators published the result of a large-scale reanalysis of over five Tb of WGS metagenomic sequences deposited in the IMG server and other public databases in search for viruses [32]. In this publication, aptly named Uncovering the Earth's Virome, samples from 3,042 geographically diverse locations were used from previously available studies. By training an algorithm to hone in on patterns in the whole dataset, they managed to predict over 125,000 partial viral genomes from within the metagenomes, effectively increasing the number of known viral genes by 16-fold, most of them phages. They also predicted which bacteria they might prey on by scanning through the associated CRISPR spacers and tRNA sequences. Interestingly, more than 30% of the intestinal and 50% of the oral viral sequences (some of the most abundant sample types) were shared by at least 10% of the sampled subjects. Furthermore, they reported HMP data had on average 3.4% and 7.4% of viral sequences in all oral and stool samples, far more than previously reported. As a corollary for this study, it was demonstrated that there is much potential for data mining in large datasets regardless of the niche, because viruses are undeniably pervasive, with viral sequences populating many of the metagenomes subjected to prokaryotic analysis. Consequently, the scientific community can learn a great deal from reanalysing data by paying attention to viruses. The success of this approach has recently been translated into a spinoff of the IMG system, the IMG/VR (at https://img.jgi.doe.gov/cgi-bin/vr/main.cgi), for virus identification and a database containing any new viral sequences obtained with this protocol, currently holding 8,389 viral isolates (6,919 of which have at least a putative host) and over 726,930 Uncultivated Viral Genomes [206, 207].

The growing interest in viromics is leading the field out of the initial exploratory phase towards a more analytic one, as the gaps in databases and taxonomy shrink thanks to large-scale projects and reanalyses. Consequently, this paves the way for new discoveries in viromics that were previously prohibitive due to their large scope. One such example was the global analysis the RNA virome by Wolf and collaborators in 2018 [208]. Since viral metagenomics has significantly increased the number of available sequences (most works had previously used pathogenic mammalian and avian RNA viruses), the group used an alignment of the protein sequence of the RNA-dependent RNA polymerase of 4,640 viruses (the only marker common to RNA viruses) for the phylogenetic analyses at a viromic scale. According to the resulting topology, they inferred that dsRNA viruses may have evolved from (+)RNA viruses in two separate events, whereas (-)RNA viruses may have evolved from dsRNA viruses. Using the tree as a scaffold they carried out phylogenomic reconstructions and detected a capsid protein that could be traced back to the last common ancestor of the main branches and evaluated the history of HGT. Even though the methods are not entirely new and more sequences may be needed to confirm or discard some of their observations, the evidence calls for a major rearrangement of the taxonomy of RNA viruses, and we have reached the point at which global phylogeny is now possible. Meanwhile, new discoveries are starting to shed light on the gaps in viral taxonomy. This was the case of a 2018 work by Shi and collaborators in which they obtained the metatranscriptome in samples from reptiles, amphibians, lungfish, ray-finned fish, cartilaginous fish and jawless fish for their RNA virome, resulting in the discovery of 214 vertebrate-associated viruses. They also managed to detect that the evolutionary history of these viruses reflects that of their hosts by comparing the phylogenies of endogenous virus elements in the animal genomes [209]. Moreover, near-future large-scale projects are expected to boost virus discovery even further, best exemplified by the Global Virome Project, a $1.2 billion worldwide multi-laboratory effort which aims to expand the systematic exploration of the virome towards viruses with zoonotic potential, in order to predict and prevent future pandemics [210]. It is estimated that over 1,670,000 different viruses, spanning 25 families, may infect mammals and birds and that between 631,000 and 827,000 of these may present a potential threat to humans. The project will start in Thailand and China but will eventually be extended to all the continents and will close the gaps in the knowledge of viral pathogens.

Undoubtedly, metagenomic datasets will continue to grow and humans alone will soon be unable to keep up with the analyses of such large volumes of data, something that is already becoming evident. Metagenomics currently relies on human-assisted bioinformatic methods but data-science methods will become increasingly common in biological sciences as the complexity of data collections ramps up and we become increasingly reliant on data mining and statistical approaches for pattern recognition. Artificial intelligence poses as the logical next step for automating the analysis of such datasets since computers can systematically optimize large-scale unbiased pattern recognition and classification tasks as long as adequate and sufficient input data is provided to train the model. In fact, basic machine learning algorithms are now routinely applied to metagenomics, as is the case of supervised learning with random forests [211]. This trend has resulted in the development of new machine learning methods which are now available for use with viromics, including deep-learning unsupervised approaches that will most probably become recurrent study tools in the near future [30, 212, 213].

**Figure 2:**
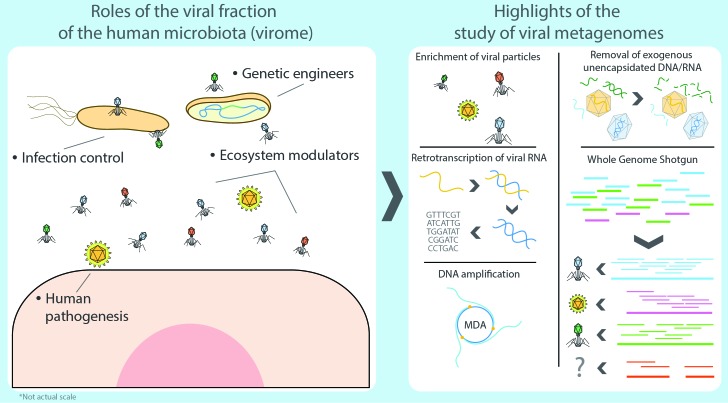
FIGURE 2: The study of the human virome. The viral fraction of the human microbiota (human virome) consists of thriving communities of viral particles, not restricted to human pathogens, but including bacteriophages and other eukaryotic viruses which actively contribute to the modulation of the ecosystem by stimulating the immune system and by directly infecting and even genetically altering certain microbial species and their genomes. Viromics, or the study of the virome and its critical impact the human host and its microbiota, relies on the isolation of such viral particles, the recovery (and amplification of their nucleic acids (multiple displacement amplification is shown) and, in the case of RNA viruses, an additional step consisting in the retrotranscription of their genomes to enable detection and sequencing. The subsequent analysis of the viral sequences depends on whole genome sequencing approaches. To this date, viromics is challenged with persistent methodological and conceptual biases and limitations that continue to be addressed with the advancement in the field of metagenomics.

The relatively young field of viromics has come far and, whatever the future may hold, it will certainly continue to thrive, for it has proven to be a uniquely versatile field. As André Lwoff once said: “viruses are viruses” and, as such, they must be defined by their own set of rules, their methods and their peculiarities. They are neither organisms nor purely inert particles, yet they are strikingly complex and some of the most effective and unpredictable genetic and ecological engineers on the planet. The history of viromics is, in fact, just the latest chapter in virology, a field characterized by innovation. Viromics is no different but is still in its infancy and will surely lead researchers towards a comprehensive ecological understanding of the human and environmental microbiota **(Figure 2)**.

## Abbreviations:

1G GA: – 1G Genome Analysis System,
CRISPR: – clustered regularly interspaced short palindromic re-peats,
DGR: – diversity-generating retroelements,
ds: – double stranded,
HBV: – Hepatitis B virus,
HGT: – horizontal gene transfer,
HMP: – Human Microbiome Project,
ICTV: – International Committee on Taxonomy of Viruses,
INSDC: – International Nucleotide Sequence Database Collaboration,
MBDC: – Molecular Biology Database Collection,
PCR: – polymerase chain reaction,
SISPA: – sequence independent single-primer amplification,
SRA: – Short Read Archive,
ss: – single stranded,
TMV: – Tobacco Mosaic virus,
WGA: – whole genome amplification,
WGS: – whole genome sequence.
